# Effect-based environmental monitoring for thyroid disruption in Swedish amphibian tadpoles

**DOI:** 10.1007/s10661-019-7590-1

**Published:** 2019-06-21

**Authors:** Gunnar Carlsson

**Affiliations:** Department of Biomedical Sciences and Veterinary Public Health, Swedish University of Agricultural Sciences, Box 7028, 750 07 Uppsala, Sweden

**Keywords:** *Rana arvalis*, *Rana temporaria*, *Bufo bufo*, Metamorphosis, Biomarkers

## Abstract

It is well-known that the metamorphosis process in amphibians is dependent on thyroid hormones. Laboratory studies have shown that several environmental contaminants can affect the function of thyroid hormones leading to alterations in the amphibian metamorphosis. The basic idea of the present study was to elucidate if the amphibian metamorphosis might be a useful tool as biomarker for effect-based environmental monitoring, examining wild tadpoles for potential thyroid hormone disruption. A laboratory test was performed to identify the responses from exposure to 6-propylthiouracil (PTU), which has a well-known mechanism on the thyroid system, on Swedish tadpoles from the *Rana* genus. This was followed by an environmental monitoring study where tadpoles of *Rana arvalis*, *R. temporaria*, and *Bufo bufo* were sampled from various sites in Sweden. Morphological data such as body weight, histopathological measurements of the thyroid glands, and environmental parameters were recorded. The results revealed that *Rana* tadpoles respond similar as other amphibians to PTU exposure, with interrupted development and increased size relative to the developmental stage. Data on some wild tadpoles showed similar features as the PTU exposed, such as high body weight, thus suggesting potential thyroid disrupting effects. However, histological evaluation of thyroid glands and pesticide analyses of the water revealed no clear evidence of chemical interactions. To a minor degree, the changes in body weight may be explained by natural circumstances such as pH, forest cover, and temperature. The present study cannot fully explain whether the high body weights recorded in some tadpoles have natural or chemical explanations. However, the study reveals that it is clearly achievable to catch tadpoles in suitable stages for the use in this type of biomonitoring and that the use of these biomarkers for assessment of thyroid disruption seems to be highly relevant.

**Electronic supplementary material:**

The online version of this article (10.1007/s10661-019-7590-1) contains supplementary material, which is available to authorized users.

## Introduction

The global extinction of species and population declines among amphibians is considered as a critical threat to global biodiversity (Whittaker et al. 2013). In Sweden, 5 of the 13 amphibian species are considered to be vulnerable (https://artfakta.artdatabanken.se/taxon/4000105). Different explanations have been suggested for the amphibian decline, including pathogens, habitat degradation, drying of wetlands associated with climate change and UV radiation (Alford and Richards 1999; Rohr and Madison 2003; Hayes et al. 2003; Whittaker et al. 2013). Pollutants including pesticide contamination of waters have been proposed as one major factor responsible for these declines (Sparling et al. 2001; Hayes et al. 2010). Amphibian reproduction and larval development might coincide both in time and place with pesticide application in agriculture. Apart from pesticides, also other chemicals which enter the environment end up in aquatic environments. Amphibians might thus be exposed to a large number of chemicals from different sources. It is therefore important to develop reliable biomarkers that reflect the amphibian health and pollutant exposure pressure. Wild population data concerning, e.g., endocrine disrupting effects in amphibians are lacking, which prevents conclusions on potential associations (Orton and Tyler 2015).

Studies have shown disruptions concerning the thyroid glands and the levels of thyroid hormones (TH) in wild animal populations including birds, fish, marine mammals, and polar bears, sometimes positively correlated to concentrations of organic pollutants in the environment (Rolland 2000; Colborn 2002; McNabb and Fox 2003; Schnitzler et al. 2008, 2012; Gabrielsen et al. 2015). Laboratory studies have also shown that several environmental contaminants can affect the thyroid gland and the function of TH, e.g., perchlorate, polychlorinated biphenyls (PCB), tetrachlorodibenzodioxine (TCDD), and chlorinated pesticides (Crisp et al. 1998; Brown et al. 2004; Crane et al. 2005). The mechanism of action causing thyroid disruption might, however, vary with different chemicals depending on the large number of potential targets in the complex regulation of TH (Gilbert et al. 2012).

It is well-known that the metamorphosis process in amphibians is dependent on TH (Brown and Cai 2007). This has resulted in the development of a guideline for detection of thyroid active compounds by the use of metamorphosing tadpoles: The Amphibian Metamorphosis Assay (AMA, OECD 2009). In this method, endpoints such as developmental stage, hind limb length, and body weight are measured to explore the impact of the tested chemicals on the thyroid system. Progress of development and growth of hind limbs are TH dependent, while general growth is independent of TH (Carlsson and Norrgren 2007; Coady et al. 2014). Additional assessment of thyroid gland histology has proven to increase the sensitivity and add more information about the mechanisms of action of the tested chemicals (Opitz et al. 2006; Carlsson and Norrgren 2007). Thus, there are well-known connections between morphological and histological responses and TH disruption in amphibian tadpoles.

Exposure to TH disrupting chemicals might thus cause interruption or retardation of the metamorphosis process (Opitz et al. 2006). In *Xenopus*, an observed pattern is a reduced development in connection with a normal growth (OECD 2009; Carlsson and Norrgren 2007). Reduced development rate is sometimes, but not always, associated with alterations in the thyroid gland such as hyperplasia and epithelial cell hypertrophy. Often, thyroid alterations are observed at lower concentrations of chemicals than those causing reductions in tadpole development, indicating a compensatory response (Carlsson and Norrgren 2007; Carlsson et al. 2019). Studies based on AMA (OECD 2009) are normally stopped around the peak of the body size, before normal tadpoles are reducing their body weight towards the end of the metamorphosis process. It can be expected that if the exposure to TH disrupting chemicals continues, the body growth increases even further, even though the developmental rate is reduced or totally interrupted, leading to abnormally large tadpoles in relatively low developmental stages, as shown by Opitz et al. (2006).

Biomarkers can be defined as biochemical, physiological, or histological indicators of exposure to, or effects of, xenobiotic chemicals (Huggett et al. 1992). Although having many advantages, biomarkers have not yet been fully exploited and incorporated in monitoring approaches (Hook et al. 2014). There is thus a need for development of robust and relevant tools which can be used for monitoring health of wild populations of animals and correlate the health to the impact from environmental pollutants. Due to the connections between TH and development, the amphibian metamorphosis might be a useful biomarker for effect-based environmental monitoring examining wild tadpoles for TH disruption. Amphibian tadpoles have generally inhabited the specific water from newly fertilized embryos throughout the metamorphosis process. Thus, an adverse effect in the metamorphosis process might be an indicator of pollutant levels and potential health impacts within the actual site. So far, however, such field studies on adverse effects of chemicals on metamorphosis are lacking (Orton and Tyler 2015).

Based on the well-studied effects of thyroid disrupting chemicals on the amphibian development, the basic idea and the overall aim of the present study was to elucidate if the amphibian metamorphosis might be a useful tool as biomarker for disruptions of the TH system, for the use in effect-based environmental monitoring. To address this, a laboratory test was performed to identify the responses from exposure to 6-propylthiouracil (PTU), which has a well-known mechanism on the TH system, on Swedish tadpoles from the *Rana* genus. This was followed by an environmental monitoring study where tadpoles of *Rana arvalis*, *R. temporaria*, and *Bufo bufo* were sampled from various sites in Sweden. Body measurements reflecting growth and development as well as histological measurements of the thyroid gland were examined. Environmental parameters, water parameters, and pesticide concentrations were recorded to reveal possible explanations to the responses.

## Materials and methods

The methods described here consisted first of laboratory tests where *Rana temporaria* and *R. arvalis* tadpoles were exposed to PTU and where unexposed tadpoles were assessed (“Background studies”); secondly, a field survey where tadpoles of *R. temporaria*, *R. arvalis*, and *Bufo bufo* were collected in waters in Sweden. Thirdly, common methods, used in both the background studies and the field survey, are described.

### Background studies

To provide a background for the interpretation of the data for the wild-collected individuals, two approaches were used in the laboratory on the closely relative species *Rana arvalis* and *R. temporaria*. The studies were performed on both species to examine if these should be treated as two groups or if they could be combined into one group in further analyses of data. *Bufo bufo* was expected to be treated as a different group, and therefore, it was not included in these studies. First, a laboratory test examining how native amphibians responds to the TH disrupting chemical PTU was performed. Secondly, sampling of untreated tadpoles was conducted to increase the number of individuals for normal thyroid epithelial cell height (ECH) throughout the development.

#### Egg collection

*Rana* eggs were collected in the beginning of May from different sites around the Uppsala area to include genetic variance in the material. All eggs from each site came from the same clutch. The eggs (50–100 per site) were kept site separated and put in aquaria with 10 L charcoal filtered tap water (20 °C) which was aerated before use. Seventy-five percent of the water was renewed three times a week (Monday, Wednesday, and Friday) throughout the whole test. After hatching, tadpoles were fed SERA micron powder and crushed flakes (SERA Vipan). Two individuals from each site were species determined at early stages using DNA, as described later.

#### Exposure to PTU

Exposure started when tadpoles had reached stage 30 (Gosner 1960) and was performed on tadpoles originating from two sites for each of the two species *Rana temporaria* and *Rana arvalis*. From each site separately, 20 tadpoles were distributed into plastic jars containing 0.5 L of charcoal filtered tap water with or without 6-propylthiouracil (PTU; Sigma-Aldrich Sweden AB). Four individuals were randomly distributed to each jar. Exposure groups included two controls (C) and one for each of low concentration (L), median concentration (M), and high concentration (H) representing 0, 1, 7, and 50 mg PTU/L, respectively. Sampling was performed at three times during the development: “Early” (stage 35–36), “Mid” (stage 37–38), and “Late” (stage 42). The individual in each jar first to reach the appropriate stage was selected for sampling, and the remaining tadpoles were left for further development and sampling at a later time. All tadpoles were thus exposed from stage 30 until the time of sampling. Tadpoles were measured and dissected for body measurements and histology of the thyroid glands, as described later. Since developmental progress varied between tadpoles from different sites and between different jars, times for start of exposure and sampling varied slightly. Excess tadpoles, not included in the experimental test, were kept in the original aquaria and sampled throughout the development to increase the data set for thyroid epithelial cell height (ECH) of “normally” developed tadpoles.

### Field survey

Wild *Rana arvalis*, *R. temporaria*, and *Bufo bufo* were sampled from various sites in Sweden. A number of tadpole measurements were recorded as well as environmental parameters, water parameters and pesticide concentrations to reveal potential indications for TH disruption.

#### Selection of monitoring waters

**Fig. 1:**
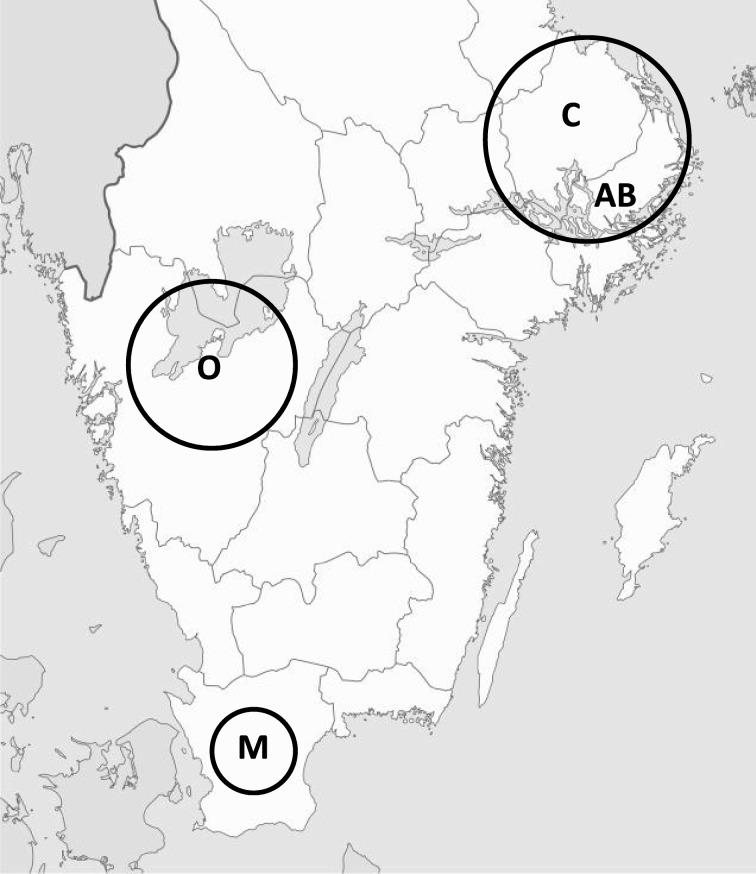
Map of the areas in Stockholm (AB), Uppsala (C), Skåne (M), and Västra Götaland (O) counties in Sweden, where tadpoles were caught

The species of interest were common frog (*Rana temporaria*), moor frog (*R. arvalis*), and common toad (*Bufo bufo*) due to their wide spread distribution in Sweden and their high relative abundance. Therefore, the areas of interest for sampling were outside localities where other more rare species of frogs and toads were present to avoid accidental catch. The counties of Uppsala, Stockholm, and Västra Götaland were chosen except for the coastal zones as well as the central part of Skåne County (Fig. 1). Information of sites where observations of one of the three species had previously been made during the reproduction period and observations of other more rare species was obtained from artportalen (https://www.artportalen.se/). Further, maps were examined for water bodies where amphibian reproduction could be suspected. Additional waters which were sighted while the actual sampling was ongoing were also included. Efforts were taken to include sites located in various landscape types, sites likely unaffected by pollution and sites influenced by activities such as roads, agriculture, landfills, and industries. The aim was to get a high number of sites with tadpoles rather than to have repeated catches for several years.

#### Tadpole sampling

A large number of sites were visited in June or July between 2010 and 2017, some of them repeatedly, up to all 8 years. The water was examined by moving a net repeatedly along the waterside. If tadpoles were found, a maximum of 11 individuals in total were collected for further analyses as well as a 250 mL water sample. Additional water samples (35 mL in 50-mL glass vials) were also collected and stored in − 18 °C for pesticide analysis. Tadpoles were measured and dissected for body measurements, as described later. For in general three individuals per site, further sampling was done for the option to purify DNA and analyze ECH. On these tadpoles, tails were frozen and stored in − 18 °C for species analyses using DNA, and the remaining bodies were fixed in phosphate-buffered formalin for histology of the thyroid glands, as described later. In total, 775 amphibian tadpoles were sampled from 56 different sites between the years 2010 and 2017. The sites where tadpoles were sampled were categorized regarding environmental parameters within 50 m from the site, at the visit, and later using web-based satellite photographs (http://kartor.eniro.se/). Presence of coniferous and deciduous trees was described separately as absent, sparse, or dense (0, 1, 2). Garden, field, pasture land, ruderal land, large road, deforestation, other water, and industrial activity were categorized as yes or no (1, 0). Water size was estimated on an ordinal scale from small to large (1–5) with approximately the following limits of water areas: 1 (< 200 m^2^), 2 (200–1000 m^2^), 3 (1000–2000 m^2^), 4 (2000–5000 m^2^), and 5 (> 5000 m^2^).

#### Water parameters

The collected water samples were measured regarding O_2_ concentration (inoLab Oxi 730, WTW GmbH, Germany) in connection with the sampling and thereafter frozen and stored in − 18 °C until further analysis. The water samples were thawed and measured regarding pH (Beckman Φ 300 pH Meter, Beckman Instruments Inc., USA) and electrical conductivity (inoLab Cond 730, WTW GmbH, Germany). Total hardness (0.1–30.0°dH), nitrate (NO_3_^−^ ≥ 5 mg/L), and ammonium (NH_4_^+^ ≥ 0.2 mg/L) concentrations were measured by the use of test stripes (Reflectoquant®, RQflex plus 10, MERCK, Germany).

#### Pesticide analyses

For selected water samples of particular interest based on deviating growth data, pesticide analysis for 103 different compounds including degradation products was performed at the Organic Risk Pollutants Laboratory, Department of Aquatic Sciences and Assessment, SLU, Uppsala, Sweden, according to a LC-MS/MS method described by Jansson and Kreuger (2010).

### Common methods

#### Tadpole measurements and dissection

Tadpoles in both the background studies and the field survey were sampled according to the same protocol. They were euthanized in tricaine methane sulfonate (MS-222; Sigma-Aldrich Sweden AB) dissolved in tap water (1 g/L) and buffered with NaHCO_3_. Subsequent to this, they were blotted dry, weighed to the nearest milligrams (body weight, BW) and stage determined according to Gosner (1960). Body length excluding tail (BL), total length including tail (TL), and hind limb length (HLL) were recorded by the use of a ruler observed under a stereo microscope. On selected individuals, the tail was frozen and stored in − 18 °C for species analysis using DNA, and/or the body was fixed in phosphate-buffered formalin for histological preparation of the thyroid gland. Additional sampling was performed although these are not reported in the present study.

#### Species identification

Separation between the two *Rana*-species was performed in two ways. At sampling, observations of the tooth rows on the posterior labium were performed if feasible, where three rows are reported to indicate *R. arvalis* and four rows indicate *R. temporaria*(Trubetskaya 2005). Alternatively, species analysis using DNA extracted from approximately 25 mg tail tissue from individual tadpoles was done according to Palo and Merilä (2003) although slightly modified (Carlsson and Tydén 2018). The aim was to do species identification on at least two individuals from each site in the field survey with the use of DNA, although this was not fully accomplished due to technical failure in some analyses.

#### Thyroid gland histology

The head from formalin-fixed specimens was processed and embedded in paraffin. Transversal serial sections (3.5 μm thick) of the head including the thyroid gland were cut and stained with hematoxylin-eosin and examined by light microscopy. Measurements of follicular epithelial cell height (ECH) were performed on digital photographs of the sections according to Carlsson and Norrgren (2007). The final value for an individual consisted of the mean value of in total 60 cell heights throughout the thyroid gland. In total, 19 tadpoles in the field survey and 53 tadpoles in the background studies were analyzed for ECH.

### Data handling and statistics

**Fig. 2:**
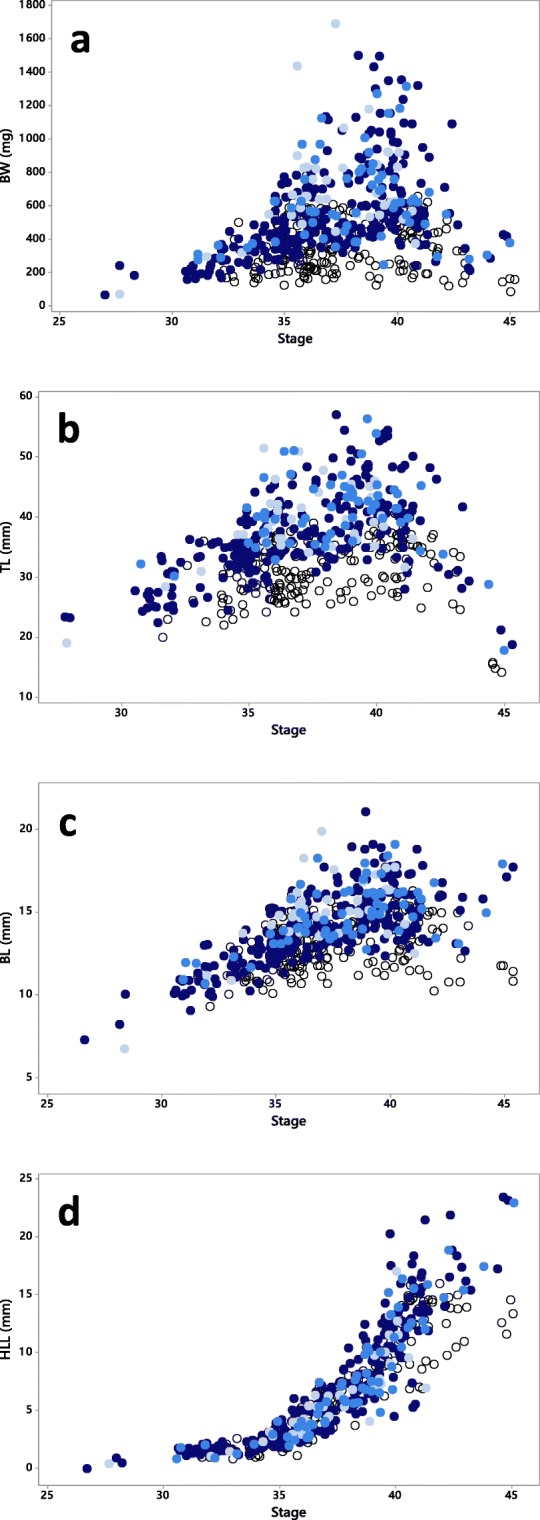
Body weight (BW, **a**), total body length (TL, **b**), body length excluding tail (BL, **c**), and hind limb length (HLL, **d**) in different developmental stages of wild collected tadpoles of *Rana arvalis* (), *Rana temporaria* (), undetermined *Rana*-species (), and *Bufo bufo* (○). Jitter is added to the graphs for separation of overlapping data points

Stages selected for further analysis of body measurements were determined in the interval from 35 to 42. At first, general linear models (GLMs) were performed on each of the response variables (BW, BL, TL, and HLL) separately (Fig. 2). First, species were used as factor and stage as covariate where the individuals determined by DNA analysis or by observation of tooth rows as either *Rana temporaria* or *R. arvalis* were included. The GLMs revealed no differences for any of the four responses between the two *Rana* species, so both these species were treated as one group, the *Rana* genus, further on in the analyses. Further, GLMs on the whole data, testing genus as factor and stage as covariate to the four responses, were performed. This revealed differences in response variables implying that *Bufo* and *Rana* should be further analyzed separately. In the GLMs, data was transformed if necessary to meet the assumptions of equal variations among groups, tested by Levene’s test. Pearson correlations were performed to reveal correlations between different response variables.

Due to poor survival rate in the experimental test of PTU, leading to lack of data, much of the interpretation of the tadpoles exposed to PTU was done including data from unexposed excess tadpoles grown beside the experimental test, and also in connection to the analyses of wild collected tadpoles. Data regarding growth, development, and ECH were analyzed for unexposed tadpoles (controls from the PTU test and excess tadpoles) for differences between species using general linear model (GLM) with species as factor and stage as covariate. Threshold values for determination of altered ECH were applied for the Rana species for the identification of data deviating from normality. These were set according to two and three times the standard deviation (SD) above and below the control mean. Pearson correlations were performed to reveal correlations between different response variables.

To enable comparisons between stages, sites, years, and species concerning the data for wild-caught tadpoles, BW data were normalized to “normal growth curves” for each genus between stages 35 to 42. Linear, quadratic, and cubic regressions were performed separately for Rana and Bufo on body weight with stage as predictor. The regression with the best fit to the data (highest *R*^2^ value and visual assessment) was selected for each genus as the “normal growth curve.” Each individual BW value was then expressed as relative to the normal growth curve for the particular stage. Threshold values for the normal range of relative BW were determined based on the whole data according to two and three times the SD above and below the mean value of log-transformed data. Graphs showing the relative BW together with the data from the experimental test of PTU were used for identifications of individuals showing similar response pattern as PTU-exposed. Such individuals were if possible measured for ECH, and water from the site was analyzed for pesticides. Also, a few thyroids of *Bufo* tadpoles considered to be normal in relative BW were measured as reference. To identify factors that might contribute to the relative body weight, Pearson (for continuous data) or Spearman (for ordinal and binary data) correlation analyses were performed on mean relative BW for each site and year, and the recorded water and environment parameters.

All data analyses were made in Minitab® 17.1.0, and the level of significance was set to *p* < 0.05.

## Results

### Exposure to PTU and excess tadpoles

Throughout the exposure period, there was a high general mortality (31, 31, 25, and 44% for controls and 1, 7, and 50 mg/L PTU, respectively) and the evaluation of ECH failed for several individuals. The mortality was, however, independent on the exposure (*p* > 0.52, Fisher’s exact test). This resulted in lack of some data points, e.g., *Rana arvalis* tadpoles, which were missing in the late sampling stage in combination with high PTU exposure. Time to reach the different stages did not differ between species and treatments except for *Rana temporaria* tadpoles exposed to the highest PTU concentration which never reached stage 42. The study was terminated after 30 days when the remaining tadpoles did not show any further development. At termination, these two individuals had interrupted their development although they apparently continued to grow.

**Fig. 3:**
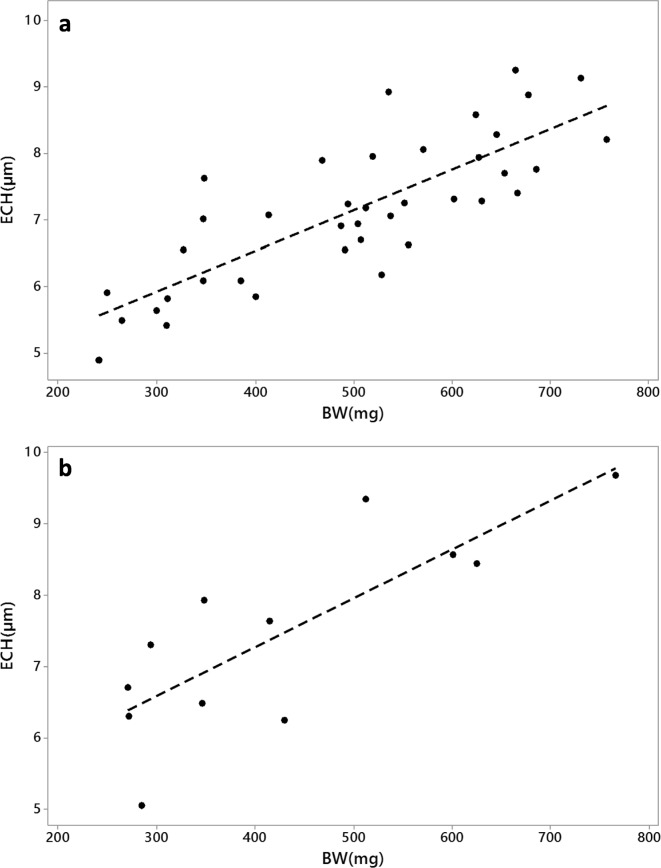
Scatterplots of thyroid epithelial cell height (ECH) versus body weight (BW) in **a** unexposed laboratory raised *Rana* tadpoles (controls and excess tadpoles) and **b** wild collected *Bufo* tadpoles

The controls in the PTU test together with excess tadpoles were combined to study normal development throughout the period of interest (stage 35 to 42). There was no difference between the two *Rana* species regarding BW (*p* = 0.358), TL (*p* = 0.507), BL (*p* = 0.843), HLL (*p* = 0.307), or ECH (*p* = 0.547) among unexposed individuals. However, stage was significantly affecting BW (*p* = 0.013), TL (*p* = 0.000), BL (*p* = 0.000), HLL (*p* = 0.000), and ECH (*p* = 0.039). There was a positive correlation recorded between stage and ECH (Pearson correlation = 0.361, *p* value = 0.033) as well as a stronger positive correlation between BW and ECH (Pearson correlation = 0.788, *p* = 0.000; Fig. 3a). Threshold values for a normal range of ECH were determined based on normal distributions of unexposed *Rana* tadpoles throughout the period. A total of 95.5% of the data (mean ± 2*SD) were expected in the range of 5.0 to 9.4 μm, and 99.7% of the data (mean ± 3*SD) were expected in the range of 4.0–10.5 μm.

### Sampled wild tadpoles—body measurements

Observing the morphological data of wild-collected tadpoles (Fig. 2), it appears that for all sampled species, there are continuous growth (BW, TL, BL) up to the stages around 40–42 and thereafter reductions both in length and weight. In contrary, HLL increases from around stage 35 and continues throughout the metamorphosis. Stages selected for further analysis of body measurements were determined in the interval from 35 to 42. Before these stages, the development progress corresponds to stages in *Xenopus*(measured according to Nieuwkoop and Faber 1994) reported to be independent of thyroid hormones (Coady et al. 2014) and later stages are associated with decreases in body weight and body length, thus complicating the interpretation of data.

In total, 29 *Rana* tadpoles were species identified both by DNA analysis and by counting tooth rows. For all of these, both methods showed the same result indicating that species identification by any of the methods could be treated as reliable. Among the wild-collected, species-determined*Rana* tadpoles, there was no difference in any of the morphological measures between *R. arvalis* and *R. temporaria* within the selected interval of developmental stages (BW, *p* = 0.147; TL, *p* = 0.292; BL, *p* = 0.196; HLL, *p* = 0.904). This implied that both species could be treated as one group, i.e., the *Rana* genus, leading to the inclusion of all undetermined *Rana* tadpoles. Corresponding analysis between *Rana* and *Bufo* tadpoles revealed differences in all recorded measures (BW, *p* = 0.000; TL, *p* = 0.000; BL, *p* = 0.000; HLL, *p* = 0.002) where *Rana* had higher values than *Bufo*. Further, the analyses showed that there were significant impacts of stage as covariate (*p* = 0.000 for all four responses). These findings implicated that the data of *Bufo* and *Rana* should be handled separately.

### Identification of deviating tadpoles

**Fig. 4:**
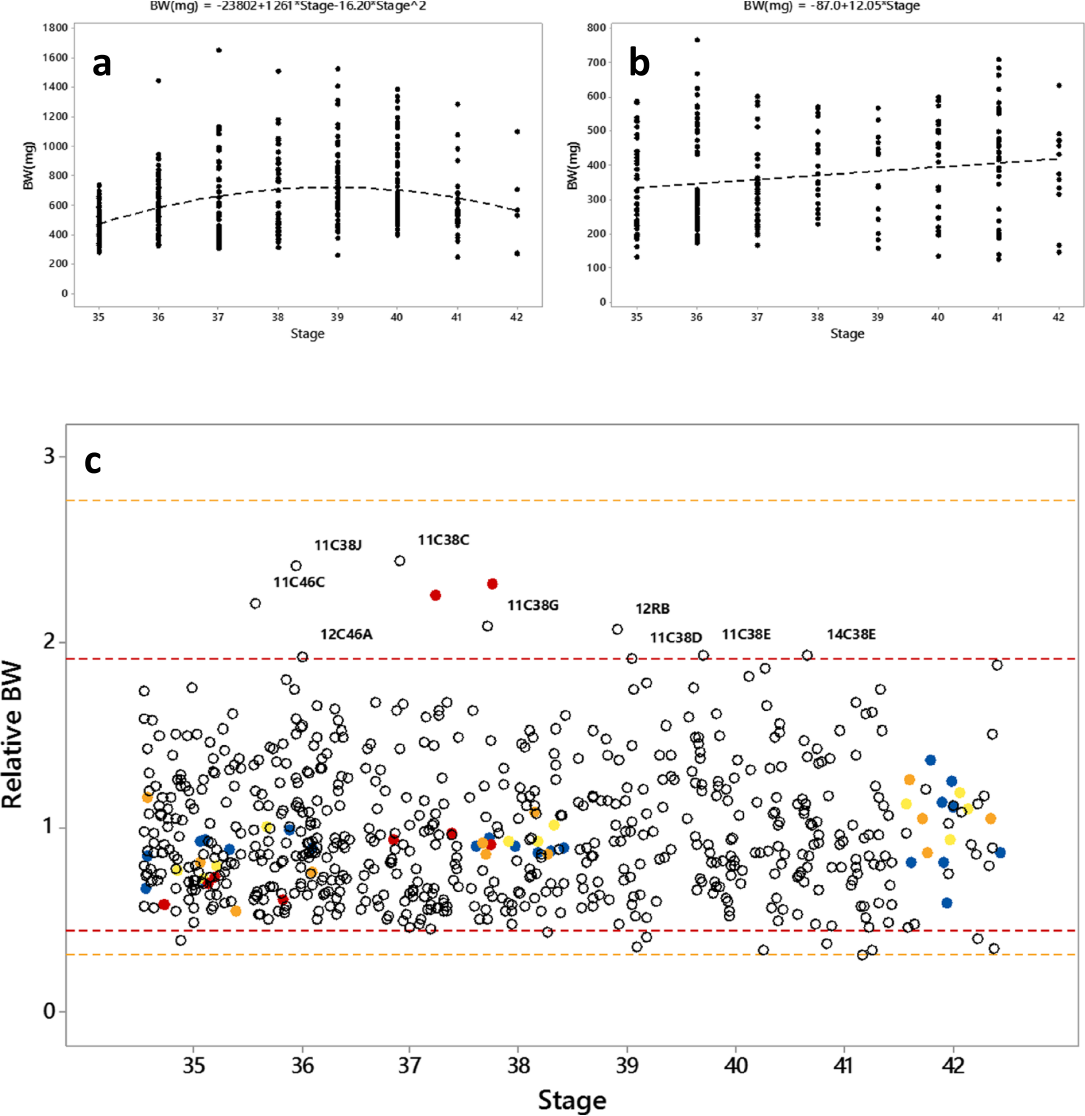
Fitted line plots (**a**, **b**) of body weight (BW) vs stage in wild collected *Rana* (**a**) and *Bufo* (**b**) tadpoles between stages 35 to 42 for extrapolation of normal body weight in the different stages. Further, a plot of BW relative to the extrapolated normal body weight (relative BW) for each individual *Rana* and *Bufo* tadpole (**c**): wild-caught (○) and tadpoles in the PTU test: controls (), 1 mg/L (), 7 mg/L (), and 50 mg/L (). Threshold lines indicating upper and lower limit of 95.5% of the data (mean ± 2*SD; red) as well as limits for 99.7% of the data (mean ± 3*SD; orange). Data points above threshold lines are marked with identification number. Jitter is added in the X-direction for separation of overlapping data points

The identification of tadpoles with features resembling tadpoles which were exposed to PTU was done by combined visualization of BW data for PTU-exposed tadpoles together with the data for wild-collected tadpoles. The BW data which were normalized to the normal growth curves between stages 35 to 42 (Fig. 4a, b) gave threshold values for the normal range of relative BW. A total of 95.5% of the data (mean ± 2*SD) were expected in the range of 0.44–1.91, and 99.7% of the data (mean ± 3*SD) were expected in the range of 0.31–2.76 (Fig. 4c). Although there are only two data points, the data indicated that tadpoles exposed to 50 mg/L PTU do not develop more than to stage 38. There were a number of tadpoles identified with high relative body weights above the 95.5% threshold values, within the same range as the tadpoles exposed to PTU that also were in similar developmental stages. It was assumed that *Bufo* respond in a similar way as *Rana* to thyroid disruption, and thus, a few of *Bufo* tadpoles were also identified with relatively high BW as compared with the majority of *Bufo* tadpoles. Some tadpoles above or just below the upper 95.5% threshold line originated from the same site and also from different years, e.g., site C38 (years 11 and 14) and site C46 (year 11 and 12). Selection of water samples for pesticide analysis was selected based on these results. Only a few pesticides were identified above LOQ, and the concentrations of these were low (Table 1).

**Table 1:** Chemical analyses for 103 pesticides including degradation products in water samples from sites identified as of particular interest. Only data of compounds identified above limit of detection (LOD) are shown. Values in italics are above limit of quantification (LOQ)

Site	Year	Compound	Concentration (ng/L)	LOD/LOQ (ng/L)
C38	2011	Terbuthylazine	1	1/2
		Terbuthylazine-desetyl	*8*	1/5
		Thiacloprid	*14*	1/2
C38	2012	Terbuthylazine-desetyl	1	1/5
		Thiacloprid	*2*	1/2
A3	2011	BAM	*37*	2/10
		Terbuthylazine-desetyl	1	1/5
C46	2011	Terbuthylazine-desetyl	1	1/5
		MCPA	*23*	5/10
C46	2012	Terbuthylazine-desetyl	1	1/5
C46	2013	Terbuthylazine-desetyl	3	1/5
		MCPA	9	5/10

*MCPA* 2-methyl-4-chlorophenoxyacetic acid, *BAM* 2,6-dichlorobenzamide

**Fig. 5:**
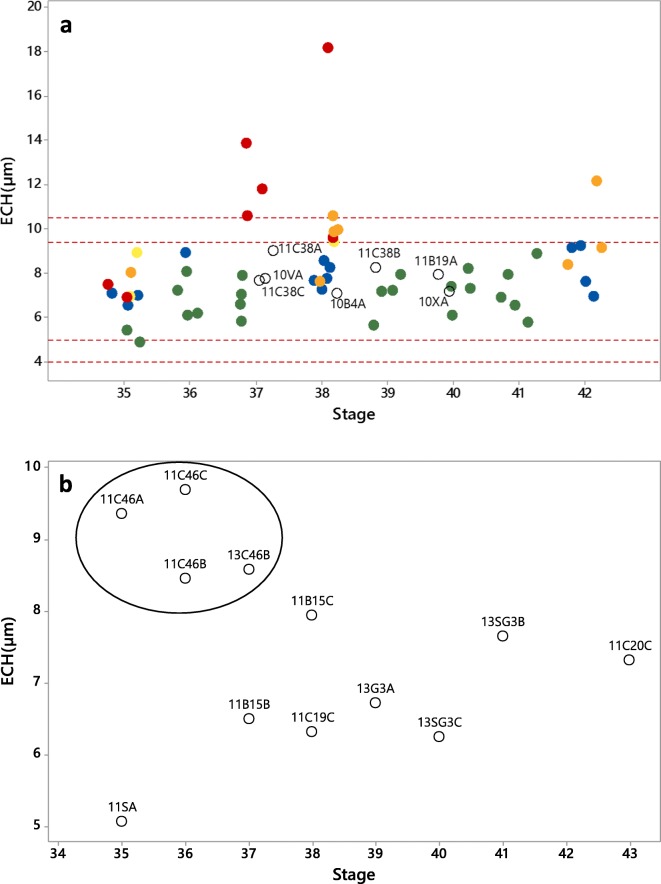
Scatterplots of thyroid epithelial cell height (ECH) versus stage in **a** *Rana* tadpoles and **b** *Bufo* tadpoles. Groups are wild-caught (○), unexposed excess tadpoles (), and tadpoles in the PTU test: controls (), 1 mg/L (), 7 mg/L (), and 50 mg/L (). Wild-caught are marked with identification number. *Bufo* tadpoles from site C46 are connected with a circle

A combination of all *Rana* tadpoles analyzed for ECH (Fig. 5a) indicates that tadpoles exposed to 50 mg/L PTU exceeded the threshold values for the unexposed. The two highest values for ECH came from the tadpoles exposed for the longest duration. Tadpoles exposed to 7 mg/L of PTU developed further to higher stages, but also some of these exceeded the threshold values for normal ECH. The wild tadpoles which were measured for ECH did not show any deviation from the normal range. *Bufo* tadpoles from site C46 seemed to have higher ECH than remaining measured *Bufo* tadpoles (Fig. 5b). Just as for untreated laboratory raised *Rana* tadpoles, there was a positive correlation in wild collected *Bufo*(Pearson correlation = 0.807, *p* = 0.002; Fig. 3b) regarding BW and ECH.

The correlation analyses revealed that coniferous trees, deciduous trees, and pH correlated negatively with the mean relative BW, while presence of field correlated positively (Table 2). All these independent variables correlated to each other, making it inappropriate to conduct a multiple regression. *R*^2^ values for linear regressions on mean relative BW and each of these factors separately were 6.3% or lower. A table of site characteristics and relative BWs is included as supplementary information (Table S1).

**Table 2:** Spearman or Pearson* correlations, including *p* values, between mean relative BW of tadpoles, and environmental and water parameters. Only variables with significant correlations to relative BW are shown

	Coniferous	Deciduous	Field	pH
Relative BW	− 0.218 *p* = 0.032	− 0.230 *p* = 0.024	0.247 *p* = 0.015	− 0.207* *p* = 0.043
Coniferous		0.736 *p* = 0.000	− 0.425 *p* = 0.000	− 0.481 *p* = 0.000
Deciduous			− 0.382 *p* = 0.000	− 0.410 *p* = 0.000
Field				0.294 *p* = 0.003

## Discussion

Most environmental monitoring programs concerning pollution impact are primarily based on the analysis of chemical substances in water, sediment, or biota. A resulting assessment is therefore based on single substances (Connon et al. 2012). By the use of biotesting, including health assessment of resident organisms using biomarkers, enables the direct measurement of toxicity independent of the number of influencing chemicals or mixture effects (Connon et al. 2012). However, the use of biomarkers has not yet been fully utilized and integrated in monitoring system (Hook et al. 2014). Inclusion of biomarkers could be a cost-effective application for effect-based environmental monitoring to increase knowledge of ecological health, environmental relevance and to link ecological and chemical information (Connon et al. 2012; Carlsson and Tydén 2018). Development of new tools that can screen multiple responses and monitor harmful effects of pollutants on ecosystem health is requested and will be important for advancing this field (Connon et al. 2012; Baker et al. 2014). In contrary to fish, amphibians are rarely used within the area of environmental monitoring for pollution. Due to the partly aquatic and partly terrestrial life of amphibians, they can migrate to wetlands without any natural water connection, far up in the water systems where reproduction might occur. The application of amphibians in environmental monitoring also includes data from wetlands without the presence of fish, since amphibian species often avoid sites inhabited by fish. The present study is thus an attempt to investigate if the amphibian metamorphosis can be useful as biomarker for monitoring of the presence of TH disrupting chemicals in the environment. The study reveals that it is clearly achievable to catch tadpoles in suitable stages for the use in this type of biomonitoring. Further, tadpoles are usually recorded in large numbers, and a minor sampling will likely not have significant impacts on future population size. The sampled species are relatively common in Sweden, but the knowledge obtained can add substantially to the information also for other more endangered species.

### Experimental test of PTU

Studies using AMA-based protocols on well-known thyroid disrupting chemicals such as PTU and methimazole have shown reductions in the development (measured on developmental stage or hind limb lengths) although body growth remains normal (Degitz et al. 2005; Carlsson and Norrgren 2007). In contrast to most such studies, the present study on *Rana* tadpoles exposed to PTU was not directed to a certain exposure time, but rather on reaching certain developmental stages. It was thus expected that the body growth would be relatively high since more time would be required to reach corresponding developmental stage as the control, or alternatively, the development would be totally interrupted in tadpoles exposed to high concentrations of PTU. This has previously been shown in *Xenopus laevis* tadpoles exposed to ethylenethiourea where time to fore limb emergence was prolonged resulting in higher whole body length at that specific stage, or in higher concentrations, total interruption of development at a lower stage (Opitz et al. 2006). Even though the experimental test in the present study was not fully successful due to high mortality, the result still indicates that in the highest concentration of PTU (50 mg/L), large tadpoles in low stages were observed. In lower concentrations of PTU, the development was similar as for the controls. Histological measurements of the ECH in the thyroid glands showed increased ECH deviating from the normal pattern in tadpoles exposed both to 7 and 50 mg/L PTU. These deviations were present both among the medium stages (37–38) of development and in the late stages (42), but not among the early stages (35–36). In a study on *Xenopus tropicalis* tadpoles exposed to PTU for 2 weeks, tadpoles exposed to 75 mg/L showed reduced development at the test ending (Carlsson and Norrgren 2007). There were no developmental retardations in tadpoles exposed to lower concentrations of PTU, but ECH were significantly increased from 5 mg/L and in higher concentrations of PTU, suggesting a compensatory regulation of the thyroid gland in response to reduced TH levels (Carlsson and Norrgren 2007). The results from the present study thus indicate that *Rana* tadpoles respond similar as other amphibians exposed to PTU. The assumption that *Rana* tadpoles exposed to thyroid disrupting chemicals can respond with high body weight and/or increased ECH seems to be correct.

### Wild-caught tadpoles

The sampling of wild tadpoles rendered a large number of data points reflecting the growth and development of the amphibian tadpoles (Fig. 2). To handle the large amount of data and keep the focus towards the issue of detection of possible thyroid disrupting effects, the analyses were concentrated on parts of the data. As explained in the result section, data analyses were performed on developmental stages from 35 to 42. In *Xenopus*, the thyroid gland becomes functional at around Nieuwkoop-Faber stage 54 (Coady et al. 2014) which approximately correspond to Gosner stage 35. The action of antagonistic thyroid disruption is therefore not relevant to study before these stages. Also, the exposure to PTU in the present study gave no indications of impact at stages 35. Except for HLL, which increases throughout the development, the other apical measurements connected to tadpole growth decrease from around stages 40–42(Fig. 2). This is partly due to remodeling of the tissues and organs, such as reductions in tail length towards the end of the metamorphosis (Ishizuya-Oka et al. 2010). Thus, the ability to distinguish between changes caused by thyroid disruption and natural progression might be complicated after stage 42.

The general pattern of the data for BW and BL throughout the developmental stages of interest (35–42) was similar, where both parameters increased with increasing stage. Also, the deviations of the two remaining *Rana* tadpoles exposed for longest time to 50 mg/L PTU were more or less equal for both BW and BL. The resolution of each measurement was higher for BW, and therefore, the remaining analyses concerning body growth were conducted using only this response. It is problematic to directly compare data of tadpoles from different species which also can be at different stages in a process where there normally are variations in response parameters, such as in the metamorphosis. To be able to make comparisons between sites, e.g., to compare a site where *Bufo* tadpoles were late in metamorphosis with a site where *Rana* tadpoles were in the intermediate phase, the BW measurements were normalized to calculated normal growth curves (Fig. 4). Applying threshold values based on the normal distribution of the relative BW, several individual observations were detected as deviating. The inclusion of the tadpoles from the PTU test in this data view showed a good fit, with the majority of the tadpoles well within the normal range of relative BW, and the two tadpoles exposed for the longest time to 50 mg/L PTU were detected as deviating (Fig. 4c). There were thus a number of wild-caught tadpoles with BWs similar as those known to be affected by a thyroid disrupting chemical. One site with clearly large tadpoles was C38, in particular in 2011. That year, abnormally large tadpoles were detected both of *R. arvalis* and *R. temporaria*, suggesting that this is likely not due to genetic reasons, but rather due to environmental causes. Although not as clear, high BWs were also recorded other years in this site. Also among *Bufo bufo*, tadpoles from site C46 deviated with higher relative BW than within the normal range. These were also detected several years, in particular 2011 and 2012. Other relative BWs above or just below threshold values were detected in site R in 2012 and site A3 in 2010. In site R, one single tadpole with high relative BW was detected. Tadpoles from this site have been caught several years, but no other deviating observation has been made. Unfortunately, there were no ability to do a histological examination of the thyroid gland for this individual.

Thyroid glands from *Rana* tadpoles from some sites close to industrial activities as well as three tadpoles from site C38 in 2011 were sectioned and measured for ECH, but these did not deviate from the normal range. Also, *Bufo* tadpoles from site C46 were measured for ECH. Since the normal pattern of ECH was evaluated only in *Rana* tadpoles, ECHs were also determined for some *Bufo* tadpoles from sites showing normal growth, to have as comparison. The ECHs from site C46 were clearly higher than from the other *Bufo* tadpoles (Fig. 5b). The fact that ECH showed a positive correlation with BW (Fig. 3b) complicates the interpretation of the high ECH in tadpoles from site C46. This might just be a normal pattern due to higher BW, caused by circumstances other than chemical exposure.

A pattern of the normal development of thyroid glands in *Xenopus* is reported to be increased in ECH throughout the metamorphosis with the maximum height between Nieuwkoop-Faber stages 62 and 64 (Dodd et al. 1976; Grim et al. 2009), i.e., close to complete metamorphosis. In contrary, the ECHs of *Rana temporaria* are reported to be more stable during the corresponding stages of development (Fox 1966). This pattern is also observed in the present study, although a weak positive correlation was recorded between stage and ECH. For identification of increased ECH, the normal inter-individual variation is of more importance than the variation between stages which suggest that the judgment can be made regardless of the developmental stage (Fig. 5). The correlation between BW and ECH is stronger and observed both among unexposed laboratory raised *Rana* tadpoles and among wild-caught*Bufo* tadpoles (Fig. 3). The increase in ECH with increasing BW does, however, not apply to the large *Rana* tadpoles in site C38, which has ECH well within the normal range despite the high BW. There are examples of chemicals that alters thyroid hormones without effects on thyroid histology (DeVito et al. 1999). Also, in amphibian studies, signs of thyroid disruption have been observed in spite of the absence of histopathological effects on the thyroid glands (Balch et al. 2006; Carlsson et al. 2007). Thus, thyroid disruption affecting BW does not necessarily have to be connected to enlarged thyroid glands and increased ECH. Although no evident alterations in ECH were detected among the investigated tadpoles in the present study, the increased BWs still might be explained by thyroid disruption.

### Chemistry

Water samples from sites identified as of particular interest were analyzed for pesticides, including degradation products. The samples came from site C38 in 2011 and 2012 as well as site C46 in 2011, 2012, and 2013 because these sites were inhabited by tadpoles identified with high relative BWs, in some cases above the threshold value. Both these sites were situated in agricultural areas. One additional water sample, A3, from 2011 was analyzed. This site was situated close to a landfill area, and tadpoles in this water also had high relative BWs, although below the threshold value. In total, a few pesticides were identified but in low concentrations (Table 1). The compounds were 2-methyl-4-chlorophenoxyacetic acid (MCPA), 2,6-dichlorobenzamide (BAM, a degradation product of dichlobenil), terbuthylazine, terbuthylazine-desetyl, and thiacloprid. A comparison with data from the regional pesticide database (http://pesticid.slu.se/epi/default.cfm, in Swedish) indicated that the measured concentrations of the pesticides in the present study were in the range of normal recordings in Sweden.

There are not much signs in literature that MCPA, BAM, or its parental compound dichlobenil should cause thyroid effects. However, in one study, *R. temporaria* and *B. bufo* tadpoles were tested for 32 days in ponds with 1 mg/L of dichlobenil (Cooke 1977). In fact, tadpoles had higher BW but were in the same stage as the controls. According to Cooke (1977), the weight increase was an indirect effect of herbicide resulting in algal blooming thus providing more feed. Interactions of thiacloprid with thyroid glands and THs have been reported in mammals and birds, although at high doses. It is also reported to cause thyroid, uterine, and ovary tumors in rodents (Tomizawa and Casida 2005). Increases both in free TH levels have also been reported in rats exposed for 30 days (Sekeroglu et al. 2014). Another neonicotinoid, imidacloprid, is reported to cause alterations in both thyroid glands as well as in plasma THs and TSH in males of the bird Red Munia (*Amandava amandava*) after oral exposure for 30 days (Pandey and Mohanty 2015). Thiacloprid toxicity in fish seems to be relatively low. Tomizawa and Casida (2005) reported LC_50_ to be 31 ppm (mg/L). Terbuthylazine has been shown to interfere with TH action in vitro. TH-dependent GH3 cell proliferation in the T-screen assay was increased from a concentration of 10^−6^ M (0.23 mg/L; Ghisari et al. 2015). Terbuthylazine is structurally related to atrazine, which has been shown to delay amphibian development in concentrations of 100 ppb (μg/L) without reducing the BW (Freeman and Rayburn 2005), thus resembling the pattern of PTU. There are thus indications that terbutylazine and thiacloprid have potential to interfere with the thyroid system although likely not at the concentrations detected in the present study. Both substances, including the metabolite terbuthylazine-desetyl, were detected at site C38 in 2011 although only at low nanograms per liter concentrations (Table 1). Further, terbutylazine-desethyl was detected in all analyzed waters in the present study. Even though it seems unlikely, it cannot be ruled out that these pesticides can be responsible, single or in combination, for the high relative BWs recorded in particular in 2011. Further studies of these compounds on thyroid effects should be promoted.

### Natural explanations for deviating observations

One major question raised is whether the deviating high BWs recorded among wild tadpoles, which are similar as detected after high exposure to PTU, might be caused by natural circumstances, independent of chemical exposure. Tadpoles have been shown to have high plasticity in the timing of the metamorphosis in response to unpredictable environments. Crowding, resource limitations, habitat desiccation, and predation have been identified as factors which may inhibit growth but stimulates development (Denver 1997). A reduction of water volume was shown to induce earlier metamorphosis in *R. temporaria* accompanied with lower size in comparison with constant high water volume, suggested to be a response to pond drying (Merilä et al. 2000). Similarly, the predator pressure can influence metamorphosis rate. A reduced time to reach metamorphosis was observed for *Bufo boreas* in the presence of predators although this was not associated with changes in size at metamorphosis (Chivers et al. 1999). There are several studies where a pattern of slow development, but metamorphosis at a large size, is recorded at low temperatures (Loman 2002). Also in *R. temporaria*, cold water temperatures lead to larger BWs at complete metamorphosis (Kuparinen et al. 2010). However, this was not observed, but rather the opposite, in a study on field data for *R. arvalis* and *R. temporaria* in Sweden (Loman 2002). The pond temperatures were not measured in the present study. It was considered hard to assess since the tadpole collection could be anytime over the day and it was assumed that temperatures showed high diurnal variations. It thus cannot be ruled out that low water temperatures are responsible for the high BWs in some sites. A limitation in the present study is that the iodine availability to the tadpoles was not measured. Low iodine intake is an important factor for development of thyroid disease (Bergman et al. 2013). However, dietary sources of iodine intake are reported to be of highest importance for aquatic vertebrates (Davis and Gatlin 1996) suggesting that measurement of iodine in water would not reflect the iodine access.

The analyses revealed some factors which individually correlated to the relative BW. However, the low *R*^2^ values indicated that these factors do not explain much of variation in the relative BW. The factors correlating to relative BW also correlate to each other. The positive correlation of deciduous and coniferous trees likely reflects that both are often present together in Swedish forests. Both these correlate negatively with fields, which are expected to be absent in forest areas. There are negative correlations between pH, and both tree types as well as a positive correlation between pH and field. This suggests that pH values are lower in forest areas while higher in more open land. Thus, there seems to be certain factors in the environment that can have impacts of the variations in the relative BW. These alone may not explain the high relative BWs detected in some sites, but there are likely a number of factors which were overlooked in the present study. There are thus no clear evidence for any thyroid disruption caused by pollutants in tadpoles from Sweden. However, the fact that tadpoles are observed which morphologically resemble those which are exposed to a thyroid disrupting compound makes it impossible to dismiss this totally.

### Biomonitoring applicability

In laboratory studies conducted on tropical frog species, changes observed in thyroid histopathology are considered clear signs for determination of disruption of the thyroid endocrine system (OECD 2009; Dang 2019). Also, increased body growth are recorded, or can be expected, as a result of exposure to thyroid disrupting compounds (Opitz et al. 2006). Both these adverse effects seem to be a fact also in the present study, where the laboratory test was conducted on amphibian species which are relevant for Swedish conditions. Based on this, the use of these two biomarkers for assessment of thyroid disruption seems to be highly relevant. The body weight seems to be a more complex endpoint to evaluate than ECH, and thus to use as biomarker. To evaluate body weights of wild-caught tadpoles where genetic variances and environmental circumstances interfere with the results is challenging. This should be seen in contrast to laboratory studies performed on sibling tadpoles under controlled environment, resulting in a minimum of variations in the data. There are other endpoints which could be applied for investigating thyroid effects in wildlife such as measurements of hormone, protein, or mRNA levels of specific mechanisms in the thyroid system which might provide a more distinct interpretation. However, also when applying measurements on mRNA levels on tadpoles from natural populations, the genetic variance complicates the interpretation (Carlsson and Tydén 2018). Nevertheless, there are needs for new tools that can monitor harmful effects of pollutants on ecosystem health (Connon et al. 2012; Baker et al. 2014), and as such, the present study is a promising attempt.

## Conclusions

A large number of anuran tadpoles from various sites around Sweden have been studied for signs of thyroid disruption. There have been tadpoles identified as deviating from the normal body weight pattern, with high body weights. These tadpoles morphologically resemble those exposed to a known thyroid disrupting compound. However, there are no clear supporting data for the fact that these findings should be caused by thyroid disruption. No histological signs of affected thyroid glands were observed among the sampled tadpoles. Further, there were no evidence of high concentrations of pesticides in the water. However, some of the identified pesticides are known to have thyroid disrupting properties in high doses. To a minor degree, the changes in body weight may be explained by natural circumstances such as pH, forest cover, and temperature, but the present study cannot fully explain whether these observations have natural or chemical explanations. The study reveals that it is clearly achievable to catch tadpoles in suitable stages for the use in this type of biomonitoring and that the use of these biomarkers for assessment of thyroid disruption seems to be highly relevant.

## Electronic supplementary material

ESM 1 (PDF 216 kb)
