# Supplementary material for: Effect-based environmental monitoring for thyroid disruption in Swedish amphibian tadpoles
Source: Environ Monit Assess. 2019 Jun 21;191(7):454. doi: 10.1007/s10661-019-7590-1 (PMC6586702; doi:10.1007/s10661-019-7590-1)
Supplement: Supplementary file 1 — (PDF 216 kb) [file 10661_2019_7590_MOESM1_ESM.pdf]

Table S1. Data on the different sites and sampled tadpoles in Sweden between 2010 and 2017. Data are presented as year and site code (Y Site), county of the site (County; AB = Stockholm, C = Uppsala, M = Skåne and O = Västra Götaland), number of tadpoles in total (n_1), number of tadpoles included in the calculation of relative BW (n_2), detected species (Sp.; B = *Bufo bufo*, A = *Rana arvalis*, T = *Rana temporaria*, R = undetermined *Rana* frog), body weight compared to the normal growth curve (RelBW) including standard deviation (SD). Environmental characteristics within 50 m radius of the site are presence of coniferous (C) and deciduous (D) trees described as absent, sparse or dense (0, 1, 2), garden (G), field (F), pasture land (P), ruderal land (Ru), large road (Ro), deforestation (Df), other water (W) and industrial activity (I) categorized as no or yes (0, 1) and water size (S) on an ordinal scale from small to large (1-5). Water measures are pH, conductivity (Cond; µS/cm), oxygen concentration (O2; %), water hardness (°dH) and ammonium (NH_4^+) and nitrate (NO_3^-) concentrations in mg/L. n.a. = not applicable; n.m. = not measured.

Y Site	County	n_1	n_2	Sp.	RelBW	SD	C	D	G	F	P	Ru	Ro	Df	W	I	S	pH	Cond	O2	°dH	NH_4^+	NO_3^-
10 B4	C	5	5	A,R	1.27	0.13	2	2	0	0	0	0	1	1	0	1	2	7.4	618	47	n.m.	n.m.	n.m.
10 V	C	5	5	A,R	1.51	0.24	2	2	0	1	0	1	0	0	0	0	2	7.0	256	43	n.m.	n.m.	n.m.
10 X	C	8	8	T,R	1.40	0.19	2	2	0	0	0	0	0	0	0	0	4	5.7	19.1	90	n.m.	n.m.	n.m.
10 R	C	8	8	A,R	0.90	0.24	2	2	0	0	0	0	0	1	0	0	3	5.7	14.3	44	n.m.	n.m.	n.m.
10 B11	C	3	0	T,R	n.a.	n.a.	1	1	0	1	1	0	1	0	0	0	4	6.7	60.0	114	n.m.	n.m.	n.m.
10 B10	C	8	7	T,R	0.72	0.15	0	2	0	0	0	1	0	0	1	0	2	7.2	1030	63	n.m.	n.m.	n.m.
10 B12	C	5	1	B,T	0.47	n.a.	2	2	0	0	0	0	0	0	0	0	4	8.9	151	109	n.m.	n.m.	n.m.
10 A3	C	8	8	A,R	1.43	0.27	2	1	0	0	0	1	0	1	0	1	2	6.6	467	13	n.m.	n.m.	n.m.
10 T	C	8	8	B	1.47	0.13	2	2	0	0	0	0	0	1	0	0	1	5.7	30.4	33	n.m.	n.m.	n.m.
10 B13	C	5	4	A,R	0.94	0.15	2	2	0	0	0	0	0	0	1	0	1	5.8	63.1	14	n.m.	n.m.	n.m.
10 B14	C	9	8	A,R	0.66	0.12	1	1	0	0	0	0	0	1	0	0	2	6.5	109	59	n.m.	n.m.	n.m.
10 B15	C	8	7	B	0.54	0.12	2	2	0	0	0	0	0	0	0	0	4	6.8	56.8	72	n.m.	n.m.	n.m.
10 A17	C	6	6	B	1.14	0.23	1	1	0	1	1	0	0	0	0	0	3	7.2	622	53	n.m.	n.m.	n.m.
10 B7	C	1	1	T	0.93	n.a.	2	2	0	0	0	0	0	1	0	0	1	7.2	157	52	n.m.	n.m.	n.m.
10 S	C	8	7	T,R	0.59	0.05	2	2	0	1	0	0	0	1	0	0	3	6.1	102	26	n.m.	n.m.	n.m.
10 B19	C	7	7	T,R	0.58	0.07	2	2	0	0	0	0	0	1	0	0	2	6.6	119	18	n.m.	n.m.	n.m.
11 C21	O	1	1	T	0.35	n.a.	2	2	0	0	0	0	0	0	0	0	2	n.m.	n.m.	n.m.	n.m.	n.m.	n.m.
11 C20	O	6	3	B	0.83	0.07	2	2	1	0	0	0	0	0	1	0	5	6.8	70.2	93	1.3	0	0
11 C19	O	11	10	B	0.70	0.09	2	2	0	1	0	0	1	0	0	0	3	6.6	233	98	3.1	0	0
11 C15	O	5	4	T,R	1.06	0.15	0	2	0	1	1	0	0	0	0	0	3	7.2	133	109	2.7	0.3	0
11 C28	O	10	9	T,R	0.68	0.13	2	2	0	0	0	0	1	0	0	0	2	6.4	564	42	1.3	0.2	0
11 C29	O	10	8	T,R	0.84	0.09	2	2	0	1	0	0	0	0	1	0	1	6.2	93.7	62	0.9	0.2	0
11 C32	O	1	0	B	n.a.	n.a.	1	1	1	1	0	1	0	0	0	0	3	n.m.	n.m.	n.m.	n.m.	n.m.	n.m.
11 C38	O	10	10	A,T,R	1.86	0.39	0	1	0	1	0	0	0	0	0	0	2	6.1	71.2	36	0.8	0.2	0
11 B4	C	1	0	A	n.a.	n.a.	2	2	0	0	0	0	1	1	0	1	2	n.m.	n.m.	n.m.	n.m.	n.m.	n.m.
11 C46	C	10	10	B	1.61	0.28	0	1	0	1	1	0	0	0	1	0	2	7.1	417	24	8.4	0.9	0
11 R	C	10	9	A,R	0.71	0.18	2	2	0	0	0	0	0	1	0	0	3	6.8	71.9	21	2.2	2.5	0
11 X	C	9	9	T,R	1.10	0.16	2	2	0	0	0	0	0	0	0	0	4	5.6	21.6	94	1.2	0	0

Y Site	County	n_1	n_2	Sp.	RelBW	SD	C	D	G	F	P	Ru	Ro	Df	W	I	S	pH	Cond	O2	°dH	NH_4^+	NO_3^-
11 V	C	1	1	A	1.09	n.a.	2	2	0	1	0	1	0	0	0	0	2	6.6	233	58	6.4	0.3	0
11 S	C	10	8	B	0.76	0.07	2	2	0	1	0	0	0	1	0	0	3	6.8	105	17	2.8	0.6	0
11 A11	C	10	10	B	0.54	0.05	2	2	0	0	0	0	0	0	1	0	2	7.5	187	55	3.6	0.2	0
11 B15	C	10	10	B	0.84	0.11	2	2	0	0	0	0	0	0	0	0	4	7.2	56.9	69	1.8	0.2	0
11 B19	C	10	10	T,R	0.68	0.08	2	2	0	0	0	0	0	1	0	0	2	6.2	97.5	22	3	0.4	0
12 E1	O	7	1	T,R	1.20	n.a.	0	2	0	1	0	1	0	0	0	0	3	7.2	118	69	2.1	0	0
12 E2	O	2	1	T,R	1.26	n.a.	0	1	0	1	0	0	0	0	0	0	3	7.1	220	65	4.1	0	0
12 C38	O	6	0	T,R	n.a.	n.a.	0	1	0	1	0	0	0	0	0	0	2	6.5	107	62	1.5	0	0
12 E9	O	10	0	A,R	n.a.	n.a.	1	1	0	1	0	1	0	0	0	0	4	9.0	348	133	6.5	0	11
12 E8	O	10	6	T,R	1.31	0.13	2	2	0	0	0	0	0	0	1	0	2	5.6	49.9	73	0.6	0	0
12 E5	O	10	1	T,R	0.79	n.a.	1	1	0	1	0	0	0	0	0	0	4	7.1	212	94	4.1	0	0
12 E3	O	10	1	B	0.95	n.a.	1	1	0	1	0	0	0	0	0	0	4	8.6	155	103	2.2	0	0
12 B4	C	4	2	A,T,R	1.25	0.18	2	2	0	0	0	0	1	1	0	1	2	7.0	591	64	7.9	0	0
12 E12	AB	10	3	T,R	1.00	0.06	2	2	0	0	1	0	0	0	0	0	1	6.9	110	58	2	0	0
12 D13	C	10	10	T,R	0.95	0.15	0	1	0	1	0	1	1	0	1	0	2	7.3	500	96	4.5	0	0
12 B14	C	10	7	B	1.47	0.22	1	1	0	0	0	0	0	1	0	0	2	6.3	60.7	27	1.1	0.2	0
12 C46	C	10	10	B	1.50	0.23	0	1	0	1	1	0	0	0	1	0	2	7.1	450	61	8.9	0	0
12 B10	C	10	9	A,R	1.01	0.14	0	2	0	0	0	1	0	0	1	0	2	7.2	513	41	11.7	0	0
12 B6	C	4	3	A,R	0.87	0.08	2	2	0	0	0	0	0	1	0	0	1	5.9	20.5	45	1.4	0	0
12 X	C	2	2	T,R	1.12	0.12	2	2	0	0	0	0	0	0	0	0	4	5.6	23.2	60	0.5	0	0
12 R	C	2	1	T,R	2.07	n.a.	2	2	0	0	0	0	0	1	0	0	3	5.1	26.2	41	0.7	0	0
12 V	C	10	9	A,R	0.75	0.09	2	2	0	1	0	1	0	0	0	0	2	6.8	142	36	2.1	0.2	0
12 T	C	10	9	A,R	0.71	0.07	2	2	0	0	0	0	0	1	0	0	1	5.8	25.7	41	0.6	0.2	0
12 S	C	6	3	T,R	1.02	0.09	2	2	0	1	0	0	0	1	0	0	3	6.8	121	43	2.4	0.2	0
12 B19	C	10	10	T,R	0.87	0.12	2	2	0	0	0	0	0	1	0	0	2	6.7	138	18	3.2	0	0
12 B15	C	10	9	B	0.84	0.08	2	2	0	0	0	0	0	0	0	0	4	7.2	4.8	65	0.6	0	0
13 L2	M	2	2	R	0.54	0.16	1	1	0	1	1	0	0	0	0	0	3	7.2	384	92	7.7	0	0
13 L7	M	10	8	B,T,R	0.57	0.15	2	2	1	1	1	0	0	0	0	0	2	7.4	106	92	0.9	0	0
13 G3	M	10	9	B	0.67	0.07	2	2	1	1	0	0	0	0	0	0	3	8.2	250	110	3.2	0	0
13 G10	M	10	7	T,R	0.52	0.08	0	1	0	1	0	0	0	0	0	0	3	7.2	270	125	4.2	0	20
13 B4	C	3	3	A	1.09	0.20	2	2	0	0	0	0	1	1	0	1	2	7.0	645	39	8.9	0.3	0
13 SG3	C	10	10	B	1.10	0.13	0	0	1	0	0	0	0	0	1	0	3	7.3	380	71	6.8	0	0
13 R	C	10	9	A,R	1.21	0.22	2	2	0	0	0	0	0	1	0	0	3	5.5	30.0	40	0.3	0	0
13 C46	C	10	10	B	1.46	0.13	0	1	0	1	1	0	0	0	1	0	2	7.1	650	32	4.5	0.2	0
13 T	C	10	10	A,R	0.77	0.08	2	2	0	0	0	0	0	1	0	0	1	5.8	33.5	56	0.4	0	0
13 V	C	8	8	A,R	1.18	0.22	2	2	0	1	0	1	0	0	0	0	2	6.7	180	13	3	0.4	0
13 B15	C	10	10	B	0.54	0.07	2	2	0	0	0	0	0	0	0	0	4	7.1	54.9	56	0.5	0	0

Y Site	County	n_1	n_2	Sp.	RelBW	SD	C	D	G	F	P	Ru	Ro	Df	W	I	S	pH	Cond	O2	°dH	NH_4^+	NO_3^-
13 B19	C	10	10	T,R	0.80	0.14	2	2	0	0	0	0	0	1	0	0	2	6.4	93.8	26	2.2	0	0
14 C38	O	9	9	T,R	1.56	0.30	0	1	0	1	0	0	0	0	0	0	2	6.4	91.5		1.4	0.5	0
14 X	C	1	1	T	0.98	n.a.	2	2	0	0	0	0	0	0	0	0	4	5.6	27.1	56	0.4	0.2	0
14 R	C	10	9	A,T,R	1.09	0.21	2	2	0	0	0	0	0	1	0	0	3	5.0	29.5	52	0.9	0	0
14 E15	AB	10	10	B	1.36	0.15	0	0	0	1	0	1	1	0	0	0	3	7.3	351	143	6.8	0	0
14 H4	AB	10	10	B	1.25	0.14	1	1	1	1	0	1	1	0	1	0	3	7.3	497	151	9.6	0	0
15 C38	O	10	8	T,R	1.36	0.18	0	1	0	1	0	0	0	0	0	0	2	6.5	81.2	25	1.3	0	0
15 B10	C	6	3	T,R	0.78	0.07	0	2	0	0	0	1	0	0	1	0	2	6.9	326	26	7.3	0.2	0
15 E12	AB	10	10	T,R	0.76	0.07	2	2	0	0	1	0	0	0	0	0	1	6.6	67.3	36	1.2	0.2	0
15 X	C	2	2	T	1.17	0.28	2	2	0	0	0	0	0	0	0	0	4	5.3	23.5	71	0.7	0.2	0
15 B15	C	6	6	B	0.90	0.07	2	2	0	0	0	0	0	0	0	0	4	6.6	39.4	48	1	0.3	0
15 B14	C	6	6	A,R	0.74	0.09	1	1	0	0	0	0	0	1	0	0	2	6.0	42.4	23	1.2	0.4	0
15 B19	C	5	5	T,R	0.85	0.15	2	2	0	0	0	0	0	1	0	0	2	6.5	50.6	30	2	0	0
15 J7	C	7	7	B	1.04	0.10	0	1	1	0	0	0	0	0	1	0	3	9.0	145	91	2.2	0.3	0
16 K11	AB	10	10	B	0.94	0.11	0	2	0	1	0	0	0	0	1	0	3	7.0	151	77	3.9	0	0
16 K12	AB	11	7	A,T,R	1.29	0.07	2	2	0	0	0	0	0	1	0	0	3	6.0	56.4	32	2.2	0.4	0
16 D7	AB	1	1	B	1.04	n.a.	0	1	0	1	0	0	1	0	0	0	3	7.4	157	112	3.8	0	0
16 J7	C	10	10	B	1.03	0.11	0	1	1	0	0	0	0	0	1	0	3	9.7	156	157	1.9	0.2	0
16 K15	AB	10	6	B	1.11	0.17	0	1	0	0	0	1	1	0	1	0	1	8.6	166	87	2.5	0.2	0
16 R	C	10	9	A,T,R	0.96	0.11	2	2	0	0	0	0	0	1	0	0	3	5.7	30.9	108	0	0	0
16 X	C	5	5	A,T	1.11	0.32	2	2	0	0	0	0	0	0	0	0	4	5.5	21.6	75	0	0	0
16 K19	AB	10	10	T,R	0.55	0.06	2	2	0	0	1	0	0	0	0	0	1	6.5	62.1	19	1	0.4	0
16 K20	C	9	4	B	0.35	0.04	1	1	0	0	0	1	0	0	0	0	2	7.8	202	69	4.3	0	0
16 B19	C	10	8	A,T,R	0.93	0.12	2	2	0	0	0	0	0	1	0	0	2	6.6	74.4	14	2	0	0
16 B15	C	10	8	B	0.62	0.05	2	2	0	0	0	0	0	0	0	0	4	6.7	59.3	29	0.9	0	0
16 B10	C	9	7	T,R	0.84	0.11	0	2	0	0	0	1	0	0	1	0	2	7.3	390	30	11.1	0	0
17 A50	C	9	7	T,R	0.65	0.15	2	1	0	0	0	0	0	0	0	0	1	7.0	112	57	0.5	0.3	0
17 A51	C	6	1	T	0.75	n.a.	2	2	1	0	1	0	1	0	0	0	1	7.2	218	24	4.7	1.4	0
17 A56	O	7	3	T,R	1.47	0.06	2	2	0	1	0	1	0	0	0	0	2	7.4	299	70	4.7	0	6
17 A53	O	3	3	A	1.35	0.08	2	2	0	0	0	1	0	1	0	0	1	6.9	112	51	1.9	0.2	0
17 A60	O	10	6	T	1.16	0.14	2	1	0	0	0	0	1	1	0	0	1	6.9	106	27	0.9	2.3	0
17 A62	O	10	9	T	1.34	0.24	2	2	0	1	0	0	1	0	0	0	2	7.0	291	37	6.6	0	0
17 H6	AB	10	9	B	0.79	0.07	0	1	0	0	0	1	1	0	1	0	3	8.9	241	139	3	0	0
17 H4	AB	10	10	B	1.43	0.19	1	1	1	1	0	1	1	0	1	0	3	7.5	341	90	2.8	0.6	0
17 K20	C	10	7	B	0.63	0.05	1	1	0	0	0	1	0	0	0	0	2	7.6	306	78	5.8	0	0
17 B19	C	9	8	T,R	0.84	0.13	2	2	0	0	0	0	0	1	0	0	2	6.6	97.5	33	2.7	0.2	0
17 D26	C	1		B	1.17	n.a.	0	0	1	1	0	1	1	0	0	0	3	7.4	90.7	75	1.2	0	0
